# Systemic and Ocular Manifestations of a Ciliopathy: A Case Report of Renal–Retinal Involvement in Senior–Loken Syndrome

**DOI:** 10.3390/jcm15052060

**Published:** 2026-03-08

**Authors:** Muzi Li, Siying Li, Yu Cao, Aimin Sun, Jinfeng Qu

**Affiliations:** 1 Department of Ophthalmology, Peking University People’s Hospital, Beijing 100044, China; lmzhsc@pku.edu.cn (M.L.); 1910301204@bjmu.edu.cn (S.L.); cy1996@bjmu.edu.cn (Y.C.); sunam@pku.edu.cn (A.S.); 2 Beijing Key Laboratory of Diagnosis and Therapy of Retinal and Choroid Diseases, Beijing 100044, China

**Keywords:** Senior–Loken syndrome, ciliopathies, retinitis pigmentosa, nephronophthisis

## Abstract

**Background**: Senior–Loken syndrome (SLS) is a rare autosomal recessive ciliopathy classically defined by the concurrence of nephronophthisis, frequently progressing to end-stage renal disease (ESRD), and retinal dystrophy, most commonly presenting as retinitis pigmentosa (RP). Given its phenotypic overlap with other renal–retinal syndromes, establishing a definitive diagnosis necessitates integrated clinical evaluation and molecular confirmation. **Case Presentation**: A 28-year-old Chinese female presented with a two-month history of binocular floaters. Her medical history was significant for ESRD of five years’ duration, managed with maintenance hemodialysis. Ophthalmic assessment revealed retinal pigment mottling along the inferior temporal arcades and generalized arterial attenuation. Spectral-domain optical coherence tomography demonstrated outer retinal thinning with loss of the ellipsoid zone at corresponding locations. Perimetry confirmed visual field constriction, and full-field electroretinography showed severely reduced rod- and cone-mediated responses. Genetic testing was performed and a pathogenic variant in the NPHP1 gene was identified. Segregation studies confirmed both parents as heterozygous carriers, consistent with autosomal recessive inheritance. Collectively, these findings established a diagnosis of SLS. **Conclusions**: This case reinforces that SLS should be considered in the differential diagnosis of any young patient exhibiting RP alongside chronic kidney disease, particularly in the setting of early-onset ESRD. It also illustrates the essential role of a coordinated, multidisciplinary approach—encompassing nephrology, ophthalmology, and genetics—in diagnosing complex ciliopathies. Genetic confirmation not only validates the clinical diagnosis but also provides a foundation for family counseling, prognostic stratification, and future eligibility for gene-specific therapeutic trials.

## 1. Background

Senior–Loken syndrome (SLS) is a rare autosomal recessive ciliopathy characterized by the combination of nephronophthisis (NPHP) and retinal dystrophy, typically presenting as early-onset retinitis pigmentosa (RP) or Leber congenital amaurosis (LCA) [1,2]. As a syndromic form of retinal degeneration, SLS exemplifies the critical role of primary cilia in both renal tubular and photoreceptor homeostasis [3,4]. The disease is genetically heterogeneous, with causative mutations identified in multiple genes encoding ciliary proteins, such as NPHP1, NPHP4, NPHP5/IQCB1, and NPHP6/CEP290.

Clinically, renal manifestations often precede or coincide with visual symptoms, with progressive chronic kidney disease typically advancing to end-stage renal disease (ESRD) within the first two decades of life. Ocular findings typically include night blindness, visual field constriction, and fundoscopic evidence of pigmentary retinopathy. An SLS diagnosis is established through a combination of clinical evaluation, imaging, electrophysiology, and genetic testing.

We report a young Chinese female patient who presented with bilateral floaters for two months and was later diagnosed with SLS based on characteristic ocular findings, a long-term history of dialysis-dependent ESRD, and confirmatory genetic testing.

## 2. Case Presentation

A 28-year-old Chinese woman presented to the ophthalmology department on 17 November 2024, with a two-month history of visual floaters in her right eye, which had progressively worsened over two weeks. The patient reported no ocular pain, redness, sensation of fullness, or increased discharge. She had been diagnosed with vitreous opacity at another hospital and treated with traditional Chinese herbal medicine for 1.5 months, without any improvement.

Her medical history was notable for intermittent fatigue beginning five years earlier, accompanied by elevated serum creatinine persisting for three months, without oliguria or generalized edema. Ultrasonography at another hospital revealed bilaterally small kidneys, and renal biopsy demonstrated ischemic kidney injury with tubular interstitial cystic changes, and a possible hereditary tubulointerstitial disorder. She was diagnosed with stage 5 chronic kidney disease and has been treated with peritoneal dialysis for five years. Regarding her family history, she denied any known family history of significant vision loss, renal disease, or nephronophthisis among her parents, siblings, or extended relatives.

**Figure 1:**
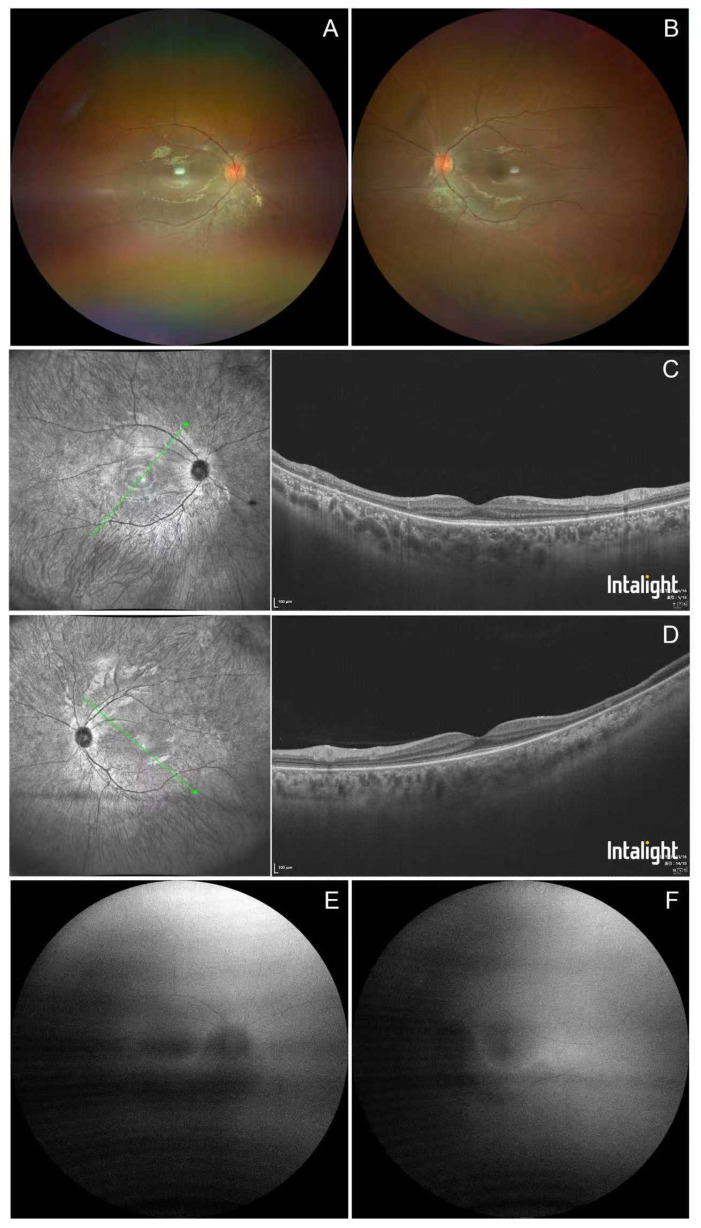
Multimodal image of a 28-year-old Chinese woman with Senior–Loken syndrome. (**A**,**B**) Color fundus photographs of the right (**A**) and left (**B**) eyes showing symmetrical, grayish-white pigment mottling along the inferior temporal vascular arcades. Note the diffusely attenuated retinal arterioles in all quadrants. (**C**,**D**) SD-OCT scans obtained through the corresponding macular regions reveal outer retinal thinning with focal loss of the ellipsoid zone at the site of pigment mottling. The ellipsoid zone remains intact at the foveal center and in the superior macular section. (**E**,**F**) AF images demonstrate band-shaped, mild hypo-autofluorescence along the inferior temporal arcades in both eyes, corresponding to the areas of pigmentary disturbance seen in panels (**A**,**B**). The arrows indicate the scan location.

**Figure 2:**
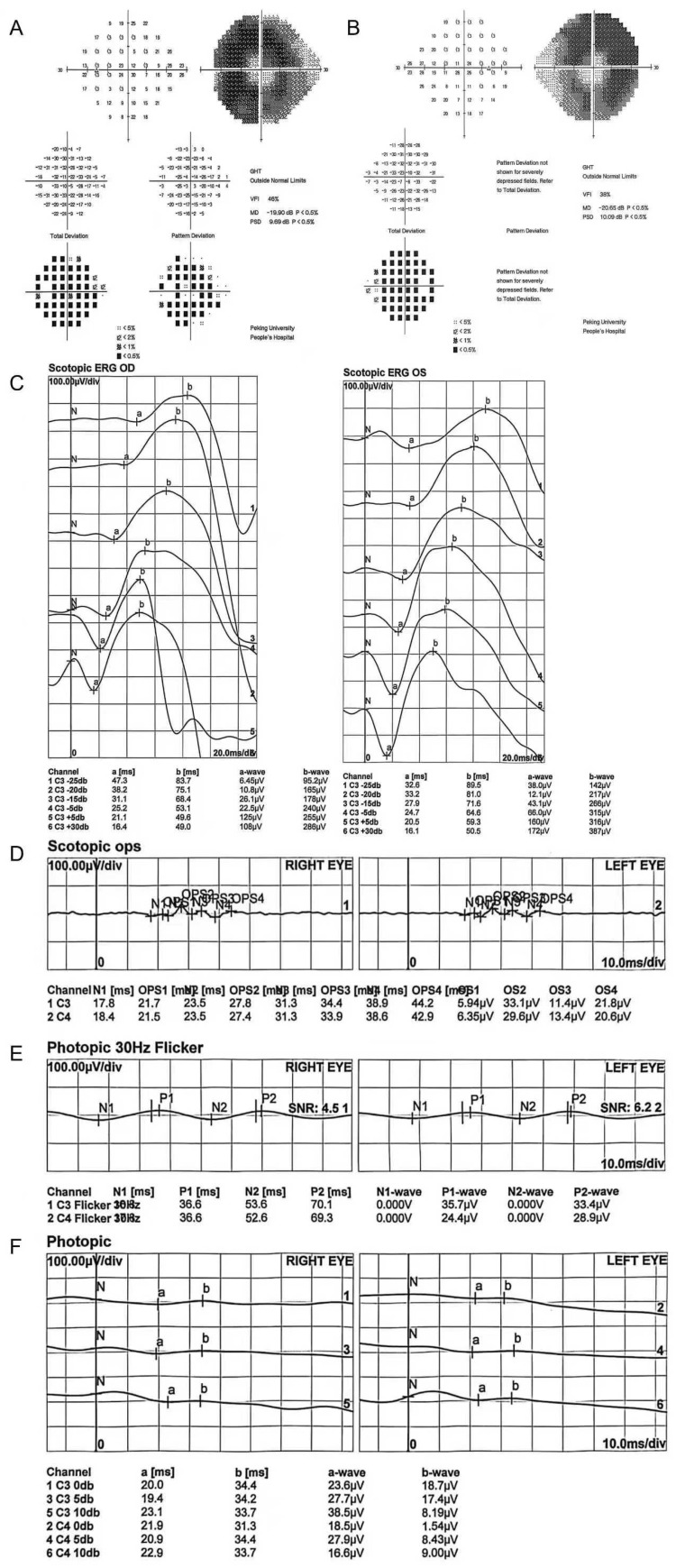
Visual field and ERG test of a 28-year-old Chinese woman with Senior–Loken syndrome. (**A**,**B**) Visual field testing showed tubular visual fields defect in both eyes. (**C**) Scotopic ERG reveals mild attenuation of a- and b-wave amplitudes with prolonged implicit times bilaterally. (**D**) Oscillatory potentials are significantly reduced in both eyes, with consequent waveform overlap reflecting severe retinal dysfunction. (**E**) 30-Hz flicker ERG shows markedly diminished waveform amplitudes. (**F**) Photopic ERG exhibits severe bilateral reduction of a- and b-waves.

We completed a comprehensive ophthalmic examination, showing that her best-corrected visual acuity was 1.0 in the right eye and 0.6 in the left eye. Her intraocular pressure was normal, and slit-lamp examination was unremarkable. Fundus examination showed symmetrical grayish-white retinal pigment mottling changes along the inferior temporal arcade in both eyes. Retinal arteries were attenuated in all quadrants bilaterally (Figure 1A,B). Optical coherence tomography (OCT) demonstrated outer retinal thinning at the inferior temporal arcade, corresponding well to the pigment mottling area (Figure 1C,D). The ellipsoid zone band disappeared at the pigment mottling area while it was preserved in the foveal center and superior section of the macula (Figure 1C,D). Fundus autofluorescence (AF) showed band-shaped mild hypo-autofluorescence along the inferior temporal arcade in both eyes (Figure 1E,F), and visual field testing showed tubular visual field defects in both eyes (Figure 2A,B). Full-field electroretinography (ERG) revealed a mild reduction in a- and b-wave amplitude and prolonged implicit times in both eyes for the scotopic response (Figure 2C), severe bilateral a- and b-wave reduction in the photopic response (Figure 2F), and a significant reduction in the oscillatory potential response (Figure 2D) and 30 Hz response (Figure 2E). These multimodal image findings point to a diagnosis of retinal pigmentosa in both eyes for this patient. Considering her ESRD, genetic testing was also performed, for which DNA was extracted from peripheral blood samples obtained from the proband and both parents. Whole-exome sequencing was performed at Chigene Corporation, followed by targeted analysis for copy number variations (CNVs) and pathogenic variants in genes associated with ciliopathies and renal–retinal syndromes. This analysis identified a homozygous deletion spanning exons 1–20 of the NPHP1 gene in the proband, and segregation analysis confirmed that both parents were heterozygous carriers of the same deletion, consistent with an autosomal recessive inheritance pattern. Given these findings, this patient was given a final diagnosis of Senior–Loken syndrome.

## 3. Discussion

The presentation of a young adult with progressive renal failure and early-onset retinal dystrophy, genetically confirmed as NPHP1-related SLS, exemplifies both the classic features and the diagnostic challenges inherent to this prototypical renal–retinal ciliopathy. These pathognomonic clinical manifestations are directly attributable to disruptions in ciliary structure and function. Specifically, the core pathophysiology of SLS lies in the dysfunction of the primary cilium. Primary cilia are microtubule-based organelles projecting from the surface of most vertebrate cells, functioning as critical signaling hubs that regulate the cell cycle, differentiation, and development [3,4]. The genes implicated in SLS (e.g., NPHP1, NPHP4, NPHP5/IQCB1, NPHP6/CEP290) encode proteins that are integral to the assembly, maintenance, and function of the ciliary apparatus [5]. In the kidney, ciliary defects lead to abnormal renal tubule epithelial cell function, resulting in NPHP, a condition histologically defined by tubular atrophy, interstitial fibrosis, and cyst formation, which inevitably progresses to ESRD in childhood or early adulthood [6]. In the eye, the connecting cilium is essential for the development and homeostasis of the photoreceptor outer segments. Photoreceptors are highly specialized retinal neurons that convert light into electrical signals through a precisely organized structure consisting of four main compartments: the outer segment (containing light-sensitive discs), the inner segment (housing metabolic organelles), the nucleus, and a synaptic axon [7,8,9]. Their function relies on a delicate structural and functional synchrony, particularly maintained by the connecting cilium—the sole physical link between the inner and outer segments—and supported by the retinal pigment epithelium, which recycles visual pigments and phagocytoses outer segment discs [3]. Disruption of any component within this system, whether in the discs, cilium, organelles, or synaptic machinery, can lead to progressive retinal degeneration [10]. Although the biological role of these primary cilia remains incompletely understood, growing evidence suggests that cilia contribute to organogenetic tissue repair and vision [11,12,13]. Consequently, mutations in SLS-associated genes trigger progressive degeneration and apoptosis of photoreceptor cells. The resulting retinal phenotype can range from severe Leber congenital amaurosis to early-onset retinitis pigmentosa with a somewhat slower progression [14], as exemplified by the current case.

The spectrum of ocular phenotypes in SLS is considerably heterogeneous. Early-onset SLS, typically presenting as LCA before the age of two, is characterized by nystagmus, amaurotic pupils, or profound vision loss, accompanied by a full-field ERG showing markedly reduced or absent responses. While the fundus may appear normal initially, optic disc pallor, vascular attenuation, and peripheral pigmentary changes occur progressively [14,15,16]. In contrast, the delayed-onset SLS typically manifests with nyctalopia and progresses to severe vision loss, mirroring the clinical course of retinitis pigmentosa. ERG in these patients is characterized by reduced a- and b-wave amplitudes and prolonged implicit times in both rod and cone responses [17,18]. The characteristic fundoscopic finding is the presence of intraretinal bone-spicule pigmentation, a hallmark of photoreceptor degeneration resulting from the migration of retinal pigment epithelium cells [17]. The specific clinical and diagnostic findings are largely dictated by the causative gene mutation and its locus [14,16], as exemplified in our patient with SLS. While NPHP1 represents one of the most frequently implicated genes in this rare ciliopathy, and large homozygous deletions spanning multiple exons have been documented, the specific deletion encompassing exons 1–20 identified here has not been reported previously, expanding the known genotypic spectrum of NPHP1-related disease. Phenotypically, the patient exhibited the classic renal–retinal manifestations of retinal dystrophy and progressive nephronophthisis consistent with established NPHP1-associated SLS, yet the adult-onset diagnosis of ocular involvement against a background of long-standing end-stage renal disease represents a distinctive clinical timeline. This contrasts with the more commonly reported early-onset ocular phenotype and highlights the phenotypic variability observed even among patients with similar deletion-based genotypes—a pattern further illustrated in prior reports where retinal involvement has ranged from childhood to late adulthood [19]. These observations collectively underscore how genetic determinants shape—but not fully constrain—the clinical expression of SLS, emphasizing the importance of considering this diagnosis even in cases of adult-onset retinal degeneration with chronic kidney disease.

In addition to Senior–Loken syndrome, several other systemic disorders are characterized by the co-occurrence of renal impairment and RP (Table 1). Mainzer–Saldino syndrome (MZSD), an autosomal recessive ciliopathy frequently associated with pathogenic variants in the *IFT140* gene, presents not only with RP and renal tubulointerstitial injury but also with characteristic skeletal anomalies, including thoracic dysplasia and short ribs [20,21]. Joubert syndrome, another autosomal recessive ciliopathy involving multiple causative genes, is strongly linked to severe retinal dystrophy, particularly when *CEP290* mutations are present, and is typically accompanied by neurological abnormalities and episodic hyperpnea/apnea [22]. Meckel–Gruber syndrome, generally considered a lethal condition, may rarely present in surviving infants with multisystem involvement, including ocular and renal anomalies [23]. Alström syndrome, an ultrarare autosomal recessive disorder caused by mutations in *ALMS1*, manifests as progressive renal dysfunction and RP, although sensorineural hearing loss represents one of its most distinguishing clinical features [24]. Furthermore, both Laurence–Moon syndrome and Bardet–Biedl syndrome—often clinically overlapping disorders resulting from ciliary dysfunction—commonly exhibit the triad of RP, hypogonadism, and cognitive impairment, underscoring the phenotypic complexity within this spectrum of ciliopathies [25] as well as the critical role of comprehensive genetic testing in achieving accurate differential diagnosis and guiding appropriate management.

Currently, no approved disease-modifying therapy exists for NPHP1-related Senior–Loken syndrome; however, therapeutic strategies for related ciliopathies illustrate potential future directions. Gene-based approaches include recombinant adeno-associated virus (AAV)-mediated gene substitution [26,27] and CRISPR/Cas9 editing—the latter overcoming AAV size constraints and showing particular relevance for large genes such as CEP290 [28,29]. RNA-targeted strategies, such as antisense oligonucleotides, offer a cost-effective alternative to modulate pathogenic transcripts, with preclinical evidence supporting their ability to restore ciliary structure and function [30,31]. While these modalities have not yet been adapted for NPHP1, emerging mechanistic insights suggest possible translational pathways. Recent studies using NPHP1-deficient human kidney organoids indicate that the Hippo signaling pathway drives renal fibrosis in this condition, and its pharmacological inhibition—potentially via repositioned agents such as verteporfin—has shown antifibrotic efficacy in vitro [32]. Thus, although patients must be counseled on the current lack of NPHP1-specific therapies, converging advances in gene, RNA, and targeted pharmacotherapies highlight a multipronged research trajectory that may eventually address this unmet need.

## 4. Conclusions

This case illustrates the classical—though frequently overlooked—fundus presentation of SLS in adult ESRD, highlights the importance of considering syndromic etiologies in patients with retinal degeneration and systemic comorbidities, and emphasizes the ophthalmologist’s integral role in the multidisciplinary management of ciliopathies. Notably, the adult-onset ocular manifestation in this patient with long-standing ESRD expands the recognized clinical timeline of SLS-related retinopathy and reinforces its inclusion in the differential diagnosis of late-presenting retinal degeneration. Given the rarity of this condition and its multisystem manifestations, early diagnosis and symptomatic management are critically important for optimizing patient outcomes. Genetic testing not only provides conclusive confirmation of the clinical diagnosis but also significantly elucidates the unified biological mechanisms underlying the syndromic presentation.

## Figures and Tables

**Table 1:** Comparative summary of clinical and genetic features in ciliopathies with renal and ocular involvement.

Feature	Senior–Loken Syndrome	Mainzer–Saldino Syndrome	Joubert Syndrome	Meckel–Gruber Syndrome	Alström Syndrome	Laurence–Moon Syndrome and Bardet–Biedl Syndrome
Primary Gene	*NPHP*	*IFT140*	*AHI1*, *CEP290*	*MKS1*, *TMEM67*, *TMEM216*	*ALMS1*	*BBS*
Inheritance	Autosomal recessive	Autosomal recessive	Autosomal recessive	Autosomal recessive	Autosomal recessive	Autosomal recessive
Key Renal Feature	Nephronophthisis (progressive)	Nephronophthisis (often early)	Nephronophthisis/renal cysts (variable)	Polycystic kidneys (large, bilateral)	Progressive renal dysfunction (interstitial fibrosis)	Renal dysplasia/CKD (common)
Key Ocular Feature	Retinal dystrophy	Retinal dystrophy	Retinal dystrophy, oculomotor apraxia	Anophthalmia/microphthalmia (severe)	Cone-rod dystrophy (very early onset, infancy)	Retinitis pigmentosa (later onset), nystagmus
Neurological Signs	Typically absent	cerebellar ataxia	‘Molar tooth sign’ in MRI, ataxia	Encephalocele/CNS malformations (lethal)	Typically absent	ataxia, poor coordination/clumsiness, learning disabilities, developmental delay
Skeletal Findings	Absent	thoracic dysplasia and short ribs	Postaxial polydactyly (occasional)	Polydactyly (postaxial)	No specific skeletal dysplasia	Postaxial polydactyly (frequent)
Other Systemic Involvement	Relatively isolated	Hepatic fibrosis	Breathing abnormalities	Hepatic fibrosis	Childhood obesity, insulin resistance, cardiomyopathy, sensorineural hearing loss	Obesity, hypogonadism, polyuria/polydipsia

## Data Availability

All data generated or analyzed during this study are included in this manuscript.

## Abbreviations

SLS	Senior-Loken syndrome
ESRD	end-stage renal disease
RP	retinitis pigmentosa
NPHP	nephronophthisis
LCA	Leber congenital amaurosis
OCT	optical coherence tomography
AF	autofluorescence
ERG	electroretinography
MZSD	Mainzer-Saldino syndrome
AAV	adeno-associated virus
